# Research on ECG signal reconstruction based on improved weighted nuclear norm minimization and approximate message passing algorithm

**DOI:** 10.3389/fninf.2024.1454244

**Published:** 2024-10-08

**Authors:** Bing Zhang, Xishun Zhu, Fadia Ali Khan, Sajjad Shaukat Jamal, Alanoud Al Mazroa, Rab Nawaz

**Affiliations:** ^1^School of Intelligent Manufacturing, Nanyang Institute of Technology, Nanyang, Henan, China; ^2^School of Mathematics and Statistics, Hainan Normal University, Haikou, China; ^3^Department of Computer Science, HITEC University, Taxila, Pakistan; ^4^Department of Mathematics, College of Science, King Khalid University, Abha, Saudi Arabia; ^5^Department of Information Systems, College of Computer and Information Sciences, Princess Nourah bint Abdulrahman University, Riyadh, Saudi Arabia; ^6^School of Computer Science and Electronic Engineering, University of Essex, Colchester, United Kingdom

**Keywords:** ECG, compressed sensing, non-local similarity, weighted nuclear norm minimization, approximate message passing algorithm

## Abstract

In order to improve the energy efficiency of wearable devices, it is necessary to compress and reconstruct the collected electrocardiogram data. The compressed data may be mixed with noise during the transmission process. The denoising-based approximate message passing (AMP) algorithm performs well in reconstructing noisy signals, so the denoising-based AMP algorithm is introduced into electrocardiogram signal reconstruction. The weighted nuclear norm minimization algorithm (WNNM) uses the low-rank characteristics of similar signal blocks for denoising, and averages the signal blocks after low-rank decomposition to obtain the final denoised signal. However, under the influence of noise, there may be errors in searching for similar blocks, resulting in dissimilar signal blocks being grouped together, affecting the denoising effect. Based on this, this paper improves the WNNM algorithm and proposes to use weighted averaging instead of direct averaging for the signal blocks after low-rank decomposition in the denoising process, and validating its effectiveness on electrocardiogram signals. Experimental results demonstrate that the IWNNM-AMP algorithm achieves the best reconstruction performance under different compression ratios and noise conditions, obtaining the lowest PRD and RMSE values. Compared with the WNNM-AMP algorithm, the PRD value is reduced by 0.17∼4.56, the P-SNR value is improved by 0.12∼2.70.

## 1 Introduction

Compressed sensing (CS) can simultaneously sample and compress signals, and accurately reconstruct the original signal from the sampled data. Once proposed, this theory has attracted widespread attention in the academic community. In order to reduce the amount of information collected by wearable ECG devices, compressed sensing technology is used to simultaneously collect and compress signals, which greatly reduces the amount of collected data, improves the energy efficiency of wearable devices, and has been widely used. Balouehestani et al. (2013) combined CS with block sparse Bayesian learning (BSBL) theory for wireless ECG acquisition, significantly reducing the sampling frequency and power consumption; Zhang et al. (2013) used CS and BSBL for remote monitoring of fetal ECG, also with the aim of reducing the sampling frequency and power consumption; Shoaran et al. (2014) used CS for the biomedical signal acquisition system of sensor arrays, which not only reduced power consumption and data volume, but also reduced the area of electrodes. Reconstructing the original signal from the compressed data is one of the core research contents of compressed sensing technology.

Reconstruction algorithms based on compressed sensing technology can be divided into three categories: (1) Convex optimization algorithms (Liu et al., 2017), which transform NP-hard *ℓ*_0_ norm problems into convex optimization problems with *ℓ*_1_ norm. (2) Greedy matching pursuit algorithm (Nguyen et al., 2017), a greedy optimization algorithm that selects the most suitable atom in each iteration based on the greedy strategy and adds it to the candidate set. (3) Bayesian reconstruction algorithms (He et al., 2010), transforming the reconstruction problem into a probability-solving problem by utilizing the prior probability distribution of the signal.

Approximate message passing (AMP) (Donoho et al., 2009), a reconstruction algorithm based on iterative thresholds, is particularly suited for real-time reconstruction in scenarios where signals such as electrocardiograms (ECGs) require rapid and efficient processing. However, the design of filters and their ability to exploit signal structure characteristics, based on AMP reconstructions, significantly impact reconstruction performance. During the research process, scholars have imposed constraints on the prior information inherent in the signal, proposing various forms of denoising models, including sparsity priors, gradient priors, non-local self-similarity priors, and low-rankness priors.

Based on the sparsity prior of images, Som (2012) employed wavelet transforms in conjunction with hidden Markov trees (HMT) for modeling and reconstruction, thus reducing computational complexity while enhancing reconstruction performance. Tan et al. (2015) applied the wavelet-domain adaptive Wiener filter to the AMP framework and proposed the AMP-Wiener algorithm. Hill et al. (2016) designed an AMP algorithm based on the Cauchy prior in the wavelet domain, which converges about twice as fast as the AMP method and improves the reconstruction quality. However, wavelet sparsity is not suitable for non-stationary natural signals. The gradient sparsity prior, which better preserves edges and textures, has attracted scholars’ attention. Wang and Liang (2015) proposed an AMP algorithm that utilizes the gradient sparsity prior to preserve image edges and texture information.

Another research direction for signal denoising is designing denoising operators that match the sparse characteristics of the target signal, where dictionary learning-based denoising is an efficient technique (Xu et al., 2021; Xue et al., 2020). The research results indicate that dictionary learning algorithms such as K-SVD (K-means singular value decomposition) (Aharon et al., 2006; Scetbon et al., 2021) and SGK (sequential generalization of K-means) have better denoising performance than denoising algorithms based on wavelet domain. Li et al. (2017) proposed a dictionary learning based AMP (DL AMP) algorithm, which integrates dictionary learning methods into the AMP algorithm to achieve denoising and achieve better image reconstruction performance than fixed transform domain denoising operators. The disadvantage is that the DL-AMP algorithm needs to train the dictionary in each iteration, and the training data entirely from the current signal to be filtered, and the number is very limited, which limits the denoising performance of the algorithm. At the same time, the dictionary training process is time-consuming, resulting in longer runtimes for DL-AMP.

The above research methods only utilize the local features of the signal, but ignore the non-local information contained in a large number of similar blocks in the signal. Metzler et al. (2016) proposed the denoising-based approximate message passing (D-AMP) algorithm, which incorporates a block-matching and 3D filtering (BM3D) denoiser (Dabov et al., 2007) that utilizes image non-local similarity priors during each iteration of the filtering process, thereby enhancing reconstruction performance. However, using non-local similarity prior requires searching for similar image blocks. The common criterion for measuring similar blocks is Euclidean distance. In the presence of noise, noise can affect the calculation of similarity between image blocks, causing a higher similarity scores between dissimilar blocks, thus impacting denoising performance.

In order to further improve reconstruction performance of compressive sensing, this paper proposes an improved weighted nuclear norm minimization algorithm for approximate message passing algorithms. In the presence of noise, dissimilar signal blocks may be grouped together, resulting in a signal block belonging to different similar block groups, so that the misclassified signal blocks obtain different denoising effects. Therefore, the weighted nuclear norm minimization (WNNM) algorithm, which averages the signal blocks after low-rank decomposition, is not suitable for obtaining the denoised signal. Theoretically, the greater the similarity between signal blocks, the lower the rank of the similar block group matrix, and the better the denoising effect. However, misclassified signal blocks will affect the low rank characteristics of the similar block group matrix, resulting in poor denoising effect. If each signal block is directly averaged to obtain the denoised signal, the details may be smoothed. Therefore, the denoised signal should be the weighted average of multiple signal blocks. Based on this, this paper improves the WNNM algorithm by proposing to use weighted averaging instead of direct averaging for the signal blocks after low-rank decomposition in the denoising process.

## 2 The background introduction

### 2.1 D-AMP algorithm

The mathematical model for compressive sensing can be expressed as:


(1) y=Φ⁢x

Signal *x* ∈ *R^N^* represents the original electrocardiogram signal, Φ ∈ *R^M×N^*(*M* ≪ *N*) is the observation matrix, and *y* ∈ *R^M^* is the obtained observation value. That is, the original ECG signal *x* is projected onto the low-dimensional space through the observation matrix Φ to obtain an *M* dimensional observation signal.

The DAMP algorithm reconstructs vector *x* ∈ *R^N^* based on the vector *y* and the measurement matrix Φ.


(2) $$x_{t+1}=D_{{\hat{\sigma }}_{t}}\,\left(x_{t}+\Phi^{T}\,z_{t}\right)$$

(3) $$z_{t}=y-\Phi \,x_{t}+z_{t-1}\,D_{{\hat{\sigma }}_{t}}^{\prime }\,\left(x_{t-1}+\Phi^{T}\,z_{t-1}\right)/M$$

(4) $${\hat{\sigma }}_{t}=\frac{{||z_{t}||}_{2}}{\sqrt{M}}$$

Where *x*_*t*_ is the t-th iteration estimate of the original signal *x*_0_, *z*_*t*_ is the residual, ${\hat{\sigma }}_{t}$ is the standard deviation of the noise, *x*_*t*_ + Φ*^T^z*_*t*_ is equivalent to the superposition of the original signal *x* and Gaussian noise, $D_{{\hat{\sigma }}_{t}}\,\left(•\right)$ represents the denoising operator acting on *x*_*t*_ + Φ*^T^z*_*t*_, making the output *x*_*t*_ closer to the original signal *x* than *x*_*t–1*_, and $D_{{\hat{\sigma }}_{t}}^{\prime }$ is the derivative of the denoiser. The denoiser plays a key role in the D-AMP algorithm, directly affecting the quality of signal reconstruction and determining the algorithm’s reconstruction performance.

### 2.2 The weighted nuclear norm minimization (WNNM) algorithm

ECG signal denoising is the process of removing noise from noisy ECG signals and restoring the original ECG signals. Let *q* = *x* + *n* where *q* is the noisy ECG signal, *x* is the original ECG signal without noise, and *n* is the noise. The ultimate goal of ECG signal denoising is to obtain an estimated value $\hat{x}$ of the original ECG signal, where $x\approx \hat{x}$.

The WNNM algorithm utilizes the similarity of signal structures and applies soft-thresholding shrinkage to singular values with different values, achieving good denoising effects. Given a noisy ECG signal *q*, let *q^i^* be a local signal block of *q*. By using block matching algorithm to search for non-local similar blocks to form matrix *Q*_*i*_, let *Q*_*i*_ = *X*_*i*_ + *N*_*i*_, where *X*_*i*_ is a matrix block of the original ECG signal without noise and is a low-rank matrix, *N*_*i*_ is a noise block. The objective function of WNNM can be expressed as:


(5) $${\hat{X}}_{i}=\arg\min_{X_{i}}\frac{1}{\sigma_{n}^{2}}\,{||Q_{i}-X_{i}||}_{2}^{2}+{||X_{i}||}_{w{,}^{*}}$$

Where $\sigma_{n}^{2}$ is the noise variance used to normalize the Frobenius norm, ||*X*_*i*_||_*w*,*_ = ∑_*i*_||*w*_*j*_σ_*j*_(*X*_*i*_)||_1_, σ_*j*_(*X*_*i*_) represents the j-th singular value of *X*_*i*_, and each item in *w* = [*w*_1_, *w*_2_, …, *w*_*n*_] is a non-negative number, corresponding to each singular value, as follows:


(6) $$w_{i}=c\,\sqrt{d}/\left(\sigma_{j}\,\left(X_{i}\right)+\varepsilon \right)$$

(7) $$\sigma_{j}\,\left(X_{i}\right)=\sqrt{\max\left(\sigma_{j}^{2}\,\left(Q_{i}\right)-d\,\sigma_{n}^{2},0\right)}$$

Where, σ_*j*_(*X*_*i*_) is the j-th singular value of *X*_*i*_. It can be observed that the larger the singular value, the smaller the weight. *c* is a constant greater than 0, *d*is the number of similar blocks, and ε is a small parameter to prevent division by zero. Perform singular value decomposition on *Q*_*i*_,*Q*_*i*_ = *U*Σ*V*.


(8) $$\varsigma_{w}\,\left(\Sigma \right)=\max\left(\Sigma_{\text{ii}}-w,0\right)$$

Where, Σ_*ii*_ is the diagonal element of the singular matrix Σ, so the solution of the objective function is obtained:


(9) $$X_{i}=U\,\varsigma_{w}\,\left(\Sigma \right)\,V$$

## 3 Proposed method

### 3.1 Improved weighted nuclear norm minimization algorithm

The WNNM algorithm has achieved good denoising results. However, when searching for non-local similar blocks, there is a possibility of misclassification by measuring the similarity of two signal blocks using Euclidean distance, which may result in grouping dissimilar blocks together and affecting the denoising effect.

In the presence of noise, the similarity between signal blocks *q^i^* and *q^j^* can be expressed as follows


(10) $$D_{q^{i}\,q^{j}}={\left(x^{i}-x^{j}\right)}^{2}+{\left(n^{i}-n^{j}\right)}^{2}+2\times \left(x^{i}-x^{j}\right)\times \left(n^{i}-n^{j}\right)$$

Where, *q^i^* = *x^i^* + *n^i^*, *q^j^* = *x^j^* + *n^j^*.

In formula (10), (*x^i^* − *x^j^*)^2^ is the distance obtained by the noise-free signal block, which is the real similarity of the signal block. (*n^i^* −*n^j^*^2^ + 2 × (*x^i^* − *x^j^*) × (*n^i^* − *n^j^*) is the error caused by noise, which may cause the Euclidean distance between signal blocks that originally have no similarity to be smaller, thus dividing them into a group. Since dissimilar signal blocks lack structural and data similarity, the resulting matrix is not a low-rank matrix, and averaging over each signal block is not suitable for denoising in the WNNM algorithm. In theory, the larger the similarity between similar blocks, the lower the rank of the matrix, Therefore, the denoised signal should be the weighted average of the signal blocks. Based on this, an improved WNNM (IWNNM) algorithm is proposed.


(11) $$X_{i}=\frac{1}{m\,\left(i\right)}\,e-\frac{r}{l}\,A_{i}$$

**FIGURE 1:**
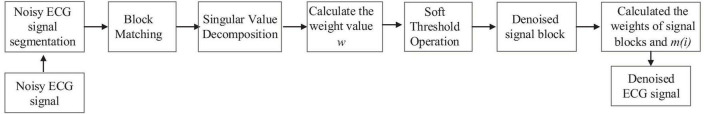
Flow chart of ECG denoising based on IWNNM.

Where $m\,\left(i\right)=\sum_{i}e-\frac{r}{l}$, *r* is the rank of the matrix ς_*w*_(Σ) and *l* is the number of rows in the matrix *Q*_*i*_. *A*_*i*_ = *U*ς_*w*_(Σ)*V* The ECG denoising process based on IWNNM is shown in Figure 1.

Firstly, the noisy ECG signal is divided into blocks. One block is selected as the reference block, while the others are candidate blocks. The block matching algorithm is used to find similar signal blocks from the candidate blocks compared to the reference block. These non-local similar blocks are arranged as column vectors to form a group of similar signal blocks. Through singular value decomposition, soft thresholding operation achieves low-rank matrix approximation, obtaining denoised signal blocks. During the process of singular value decomposition, different weight values are assigned to signal blocks based on the rank of similar signal block groups. The denoised signal is obtained by taking the weighted average of the denoised signal blocks. The denoising process of the IWNNM algorithm is shown in Algorithm 1.

**Algorithm 1:** Improved WNNM (IWNMM) algorithm denoising process.

Input noisy signal *q*, number of iterations *K* Initialize ${\hat{x}}_{0}=q,q_{0}=q$ for *t* = 1*:K* do Regularize $q_{t}={\hat{\text{x}}}_{t-1}+\delta \,\left(q-{\hat{q}}_{t-1}\right)$, and divide vector *q*_*t*_ into different signal blocks. For each sub-block *q^i^* do Find its similar sub-block group *Q*_*i*_. Singular Value Decomposition[*U*,Σ,*V*] = *svd*(*Q*_*i*_) Where *A*_*i*_ = *U*ς_*w*_(Σ)*V* Estimate obtained: ${\text{X}}_{i}=\frac{1}{m\,\left(i\right)}\,e-\frac{r}{l}\,{\text{A}}_{i}$, ，其中$m\,\left(i\right)=\sum_{i}e-\frac{r}{l}$ end Aggregate X_*i*_ to obtain a clean signal ${\hat{x}}_{t}$ End Output: ${\hat{x}}_{t}$

### 3.2 ECG signal reconstruction based on improved weighted nuclear norm minimization (IWNNM-AMP) and approximate message passing algorithm

The improved weighted nuclear norm minimization algorithm is combined with the approximate message passing algorithm to reconstruct the compressed ECG signal. The algorithm flow is as follows:

For the noisy electrocardiogram data *q* = *x* + Φ*^T^z*, divide the noisy ECG data into signal blocks of specified size. Let *q^i^* be the local block of *q* and serve as the reference block. The others are candidate blocks. Through block matching algorithm, similar signal blocks are found from candidate blocks that match the reference block. These non-local similar blocks are arranged into column vectors to form a matrix *Q*_*i*_, where the similarity between two signal blocks is expressed by formula (12):


(12) $$D_{q^{i}\,q^{j}}={||q^{i}-q^{j}||}_{2}^{2}\,$$

Where $||•|{|}_{2}^{2}$ represents the Euclidean distance between two signal blocks. If the similarity between two signal blocks is less than or equal to a preset threshold, they are considered to be similar.

Assume that *X*_*i*_ is the original ECG signal matrix block without noise, and *N*_*i*_ is the noise block, then *Q*_*i*_ = *X*_*i*_ + *N*_*i*_. By using the low-rank matrix approximation method to estimate *X*_*i*_ from *Q*_*i*_. Let *Q* = [*Q*_1_, *Q*_2_, …, *Q*_*n*_], denoise each similar block group *Q*_*i*_, then obtain the denoised signal $\hat{x}$. By using the IWNNM algorithm to denoise matrix *Q*_*i*_, the objective function of denoiser $D_{{\hat{\sigma }}_{t}}\,Q_{i}$ is as follows:


(13) $$X_{i}=\arg\min_{X_{i}}\frac{1}{\sigma_{n}^{2}}\,{||Q_{i}-X_{i}||}_{2}^{2}+{||X_{i}||}_{w{,}^{*}}$$

Denoise each similar block group *Q*_*i*_ to obtain the corresponding *X*_*i*_, aggregate all denoised matrix blocks to obtain *X* = [*X*_1_, *X*_2_, …, *X*_*n*_], and take the weighted average of the denoised signal blocks to obtain the denoised signal *x*_*t* + 1_.

By using the Monte Carlo method (Ramani et al., 2008) to obtain the derivative of $D_{{\hat{\sigma }}_{t}}$ (⋅), we have


(14) $$D_{{\hat{\sigma }}_{t}}^{\prime }\,\left(q_{t}\right)=E\,\left(\frac{b}{\tau }\,D_{{\hat{\sigma }}_{t}}\,\left(q_{t}+\tau \,b\right)-D_{{\hat{\sigma }}_{t}}\,\left(q_{t}\right)\right)$$

Where *b* ∈ ℜ*^N^* is a random vector that conforms to the standard normal distribution, τ = ||*q*_*t*_||∞/1000. Let $d_{t}=z_{t-1}\,D_{{\hat{\sigma }}_{t}}^{\prime }\,\left(x_{t-1}+\Phi^{T}\,z_{t-1}\right)/M$, then


(15) $$z_{t}=y-\Phi \,x_{t}+d_{t}$$

(16) $${\hat{\sigma }}_{t}=\frac{{||z_{t}||}_{2}}{\sqrt{M}}$$

Where *x*_*t*_ is the t-th iteration estimate of the reconstructed electrocardiogram signal *x*_0_, *z*_*t*_ is the residual, ${\hat{\sigma }}_{t}$ is the standard deviation of the noise, and $D_{{\hat{\sigma }}_{t}}^{\prime }$ is the derivative of the denoiser. The ECG signal reconstruction based on improved weighted nuclear norm minimization and approximate message passing algorithm is shown in Algorithm 2.

**ALGORITHM 2:** IWNNM-AMP algorithm process.

Input*y*, number of iterations *K* for *t* = 0, 1, ⋯, *K*do (1) *q*_*t*_ = *x*_*t*_ + Φ*^T^z*_*t*_, Split vector *q*_*t*_ into blocks and search for similar block groups *Q*_*i*_ for each signal block *q^i^*. (2) Use Algorithm 1 to implement denoising and get *x*_*t+1*_. (3) Calculate residuals $z_{t+1}=y-\Phi^{T}\,x_{t+1}+\frac{1}{M}\,z_{t}\,D_{{\hat{\sigma }}_{t}}^{\prime }\,\left(q_{t}\right)$ (4) Estimate the noise variance $\sigma^{t}=\frac{1}{\sqrt{M}}\,{||z_{t}||}_{2}$ end Output:${\hat{x}}_{t+1}$

## 4 Experiments and discussion

### 4.1 Experimental setup

To verify the reconstruction effect of the IWNNM-AMP algorithm on ECG signals, data from the MIT-BIH Arrhythmia Database (MITDB) was used to evaluate the performance of the proposed algorithm. This dataset contains 48 ECG signal records, each with two leads (MLII and V5), lasting approximately 30 min, and sampled at 360 Hz. This experiment uses MLII lead data, selects 113th ECG signal with more obvious noise for the experiment, and uses MATLAB R2020b software. In the experimental part, the reconstruction performance of the IWNNM-AMP algorithm is compared with that of the AMP algorithm based on wavelet threshold method (Liu et al., 2010) (WAVE-AMP), the AMP algorithm based on empirical mode decomposition (Xi et al., 2014) (EMD-AMP), the AMP algorithm based on WNNM (WNNM-AMP), the AMP algorithm based on NLM (NLM-AMP), and the AMP algorithm based on LRA-SVD (LRA-SVD-AMP) (Guo et al., 2016) under different noise conditions and compression rates. In the IWNNM-AMP, WNNM-AMP, and NLM-AMP algorithms, the block size is set to 32×1 and the step size is set to 1. The WAVE-AMP and EMD-AMP algorithms use the settings specified in the corresponding references. All tests are conducted in 100 independent experiments, and the results are averaged.

### 4.2 Evaluation of performance metrics

The reconstruction performance is highly correlated with the compression ratio CR, which is defined as:


(17) $$\text{CR}=\frac{N}{M}$$

Where *M* is the length of the measured signal and *N* is the length of the original ECG.

Percentage root-mean-squared difference (PRD) is used to measure the distortion level of the reconstructed signal $\hat{x}$ relative to the original signal *x*. PRD is defined as:


(18) $$\text{PRD}=\frac{{||\hat{x}-x||}_{2}}{{||x||}_{2}}\times 100\%$$

Root mean square error (RMSE) is used to compare *x* and $\hat{x}$ based on errors.


(19) $$\text{RMSE}=\frac{{||\hat{x}-x||}_{2}}{\sqrt{N}}$$

Rebuilding signal-to-noise ratio (SNR) and Peak Signal-to-Noise Ratio (P-SNR) are used to estimate the quality of the recovered signal. SNR is defined as:


(20) $$\mathrm{SNR}\,\left(\mathrm{dB}\right)=10\,\log_{10}\left(\frac{{||x||}_{2}^{2}}{{||x-\hat{x}||}_{2}^{2}}\right)$$

P-SNR is defined as:


(21) $$\text{P-SNR}\,\left(\mathrm{dB}\right)=10\,\log_{10}\left(\frac{\max{\left(x\right)}^{2}N}{{||x-\hat{x}||}_{2}^{2}}\right)$$

### 4.3 Comparison and analysis of experimental results

#### 4.3.1 The impact of different iteration numbers on reconstruction results

**FIGURE 2:**
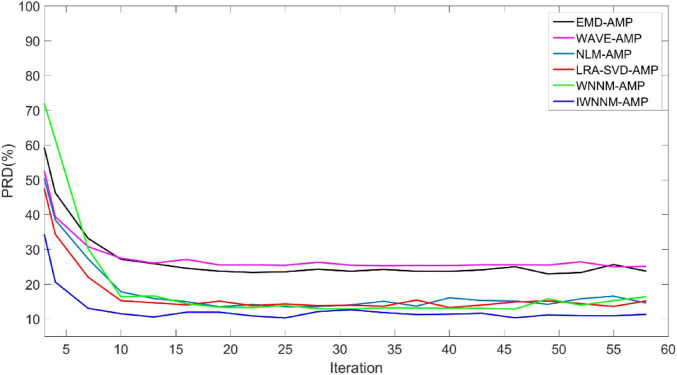
Comparison of PRD values with iteration numbers for six reconstruction algorithms under noise-free conditions.

The AMP algorithm has the characteristic of fast convergence. By comparing the PRD∼number of iterations of the six algorithms, the convergence of the six algorithms is compared. Figure 2 shows the curves of the PRD values of the six algorithms changing with the number of iterations when there is no measurement noise and the compression ratio CR = 5.12. In the absence of observation noise, all six algorithms have good convergence.

The PRD value of the IWNNM-AMP algorithm is lower than that of the EMD-AMP, WAVE-AMP, WNNM-AMP, NLM-AMP and LRA-SVD-AMP algorithms, and the convergence speed is faster. After the number of iterations reaches 30, the PRD values obtained by the six algorithms change little. Therefore, this article will use the number of iterations *K* = 30 as an example to demonstrate the simulation results.

**FIGURE 3:**
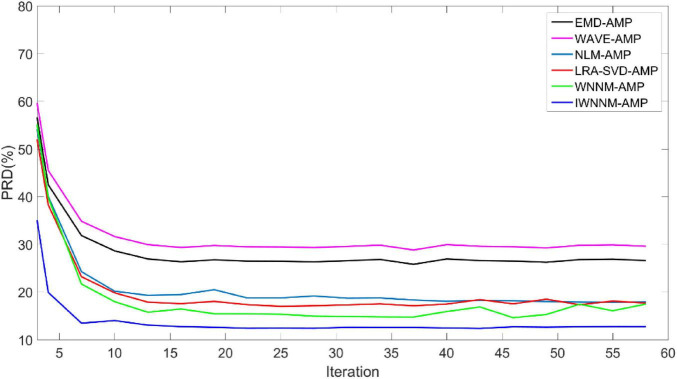
Comparison of PRD values with iteration numbers for six reconstruction algorithms under the condition of noise variance of 15.

Figure 3 shows the curves of the PRD values of the six algorithms changing with the number of iterations when the measurement noise variance is 15 and the compression ratio CR = 5.12. In the presence of observation noise, all six algorithms have good convergence performance, and the reconstruction performance of the IWNNM-AMP algorithm is better than that of the other five methods.

#### 4.3.2 The impact of different compression rates on reconstruction results

**FIGURE 4:**
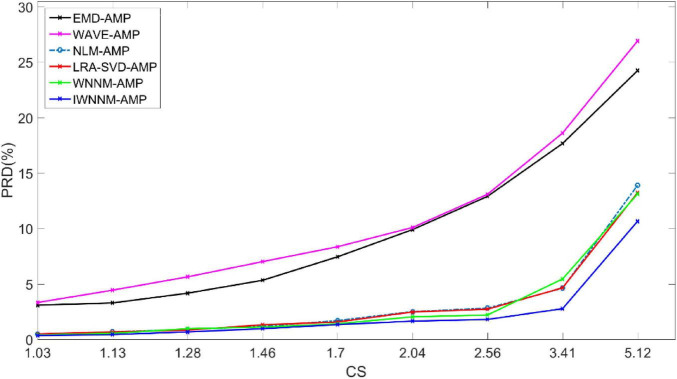
Comparison of PRD values with compression rate variation for six reconstruction algorithms under noise-free conditions.

When the compression ratio varies from 1.03 to 5.12, the impact of the compression ratio CR on the performance of the six reconstruction algorithms is analyzed. Figure 4 shows the variation of PRD values of the six algorithms with compression rate when measurement noise is not considered and the number of iterations is 30. The experimental comparison results show that under different compression rates, the PRD value of the proposed method are generally the lowest, indicating that compared with other methods, the proposed method has better objective reconstruction performance. At the same time, as the CR value increases, the PRD of the reconstruction results of various methods gradually improves. Meanwhile, as the CR value increases, the PRD of the reconstruction results of the six algorithms gradually increases.

When the CR is 1.7–2.04, the maximum PRD difference between the IWNNM-AMP algorithm and the EMD-AMP, WAVE-AMP, NLM-AMP, LRA-SVD-AMP and WNNM-AMP algorithms is 8.45. When the CR is 5.12, the IWNNM-AMP algorithm has the largest difference in PRD compared to the WAVE-AMP algorithm. The PRD of the proposed method is 13.59, 16.27, 3.24, 2.57, and 2.46 lower than that of the EMD-AMP, WAVE-AMP, NLM-AMP, LRA-SVD-AMP, and WNNM-AMP algorithms, respectively. As the CR value increases, the reconstruction performance of the IWNNM-AMP algorithm is better than that of the comparison algorithm.

**FIGURE 5:**
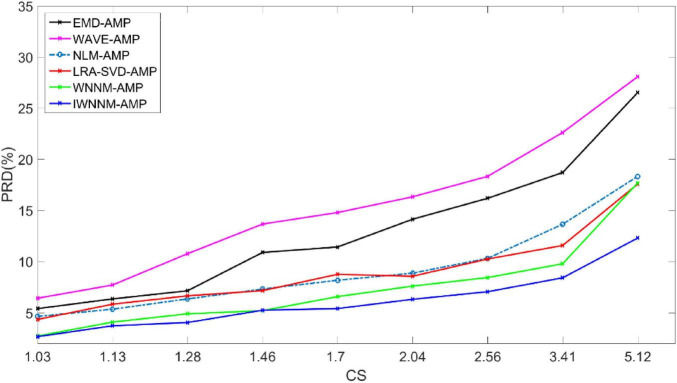
Comparison of PRD values with compression ratio for six reconstruction algorithms under the condition of noise variance of 15.

Figure 5 shows the curve of PRD values versus compression ratio for six algorithms with a measurement noise variance of 15 and 30 iterations. In the presence of observation noise, the reconstruction performance of the IWNNM-AMP algorithm is better than that of the other five methods. At the same time, as the CR value increases, the reconstruction performance of the IWNNM-AMP algorithm is better than that of the comparison algorithms.

#### 4.3.3 The impact of different noise levels on the reconstruction results

To verify the denoising effect of the algorithm under different noise levels, Gaussian white noise with noise intensities of 15 dB, 20 dB, and 25 dB was added to the 113th ECG signal, and the performance metrics of the six algorithms under different noise conditions were calculated.

Table 1 shows the average values of reconstructed PRD of the six algorithms under three different noise conditions when the compression ratio CR = 5.12 and the number of iterations is 30. The average experimental data show that when the noise variance is 15, compared with EMD-AMP, WAVE-AMP, NLM-AMP, LRA-SVD-AMP and WNNM-AMP, the PRD/RMSE values of the proposed method are lower than 14.296/0.044, 17.498/0.053, 6.389/0.014, 4.660/0.010 and 4.564/0.013, respectively. SNR/PSNR values were higher than 6.625/6.881, 7.605/7.605, 3.419/3.313, 2.741/2.778 and 2.704/2.706, respectively.

**TABLE 1:** Reconstruction performance of different algorithms under different noise.

噪音水平	重构算法	PRD	RMSE	SNR	PSNR
15 dB	EMDD-AMP	26.792	0.082	11.439	28.218
	WAVE-AMP	29.994	0.091	10.459	27.494
	NLM-AMP	18.885	0.052	14.645	31.786
	LRA-SVD-AMP	17.156	0.048	15.323	32.321
	WNNM-AMP	17.060	0.051	15.360	32.393
	IWNNM-AMP	12.496	0.038	18.064	35.099
20 dB	EMDD-AMP	25.393	0.077	11.905	28.684
	WAVE-AMP	28.230	0.085	10.985	28.282
	NLM-AMP	13.293	0.040	17.527	34.520
	LRA-SVD-AMP	13.520	0.041	17.380	34.378
	WNNM-AMP	12.058	0.036	18.374	35.409
	IWNNM-AMP	11.887	0.036	18.498	35.533
25 dB	EMDD-AMP	23.764	0.072	12.481	29.479
	WAVE-AMP	26.308	0.080	11.598	28.377
	NLM-AMP	9.171	0.027	20.61	37.75
	LRA-SVD-AMP	11.220	0.034	18.992	36.133
	WNNM-AMP	9.306	0.028	20.624	37.783
	IWNNM-AMP	8.961	0.027	20.952	38.111

The average experimental data show that when the noise variance is 20, compared with EMD-AMP, WAVE-AMP, NLM-AMP, LRA-SVD-AMP and WNNM-AMP, the PRD/RMSE values of the proposed method are lower than 13.506/0.041, 16.343/0.049, 1.406/0.004, 1.633/0.005 and 0.171/0, respectively. SNR/PSNR values were higher than 6.593/6.849, 7.513/7.251, 0.971/1.013, 1.118/1.155 and 0.124/0.124, respectively.

The average experimental data show that when the noise variance is 25, compared with EMD-AMP, WAVE-AMP, NLM-AMP, LRA-SVD-AMP and WNNM-AMP, the PRD/RMSE values of the proposed method are lower than 14.785/0.045, 17.347/0.053, 0.210/0, 2.259/0.007 and 0.345/0.001, respectively. SNR/PSNR values were higher than 8.471/8.632, 9.354/9.734, 0.342/0.361, 1.960/1.978 and 0.328/0.328, respectively.

The paired *t*-test method was used to statistically analyze the PRD values of different algorithms in Table 1, and *P* < 0.05 was considered to be statistically significant. *t*-tests were conducted on the PRD values of the IWNNM-AMP algorithm and other algorithms at different noise levels, and the results are shown in Table 2.

**TABLE 2:** *t*-test for PRD values of different algorithms.

Algorithm	EMDD-AMP	WAVE-AMP	NLM-AMP	LRA-SVD-AMP	WNNM-AMP
*P*-value	0.0005	0.0003	0.426	0.235	0.538

From the results in Table 2, it can be seen that compared with the EMDD-AMP and WAVE-AMP algorithms, the reconstruction effect of the IWNNM-AMP algorithm is significantly improved (*P* < 0.001). This is because empirical mode decomposition and wavelet denoising require an understanding of the frequency domain characteristics of the signal, while the IWNNM-AMP algorithm is a blind denoising method that utilizes the structural features of the signal to achieve denoising, making it more advantageous in reconstructing compressed signals. Compared with the NLM-AMP algorithm, LRA-SVD-AMP algorithm, and WNNM-AMP algorithm, the IIWNNM-AMP algorithm has lower PRD values by 6.389, 4.66, and 4.56, respectively, when the noise variance is 15. However, when the noise variance is 20 and 25, the PRD values are relatively close (*P* > 0.05).

**FIGURE 6:**
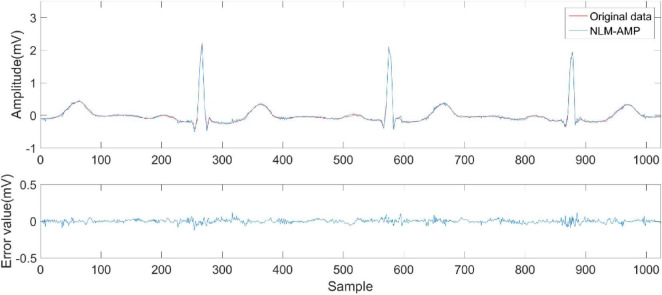
Reconstruction results of NLM-AMP algorithm.

**FIGURE 7:**
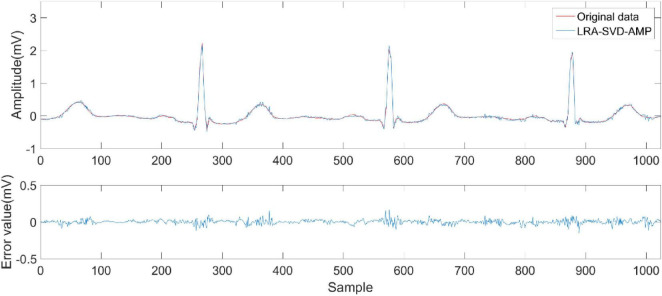
Reconstruction results of LRA-SVD-AMP algorithm.

**FIGURE 8:**
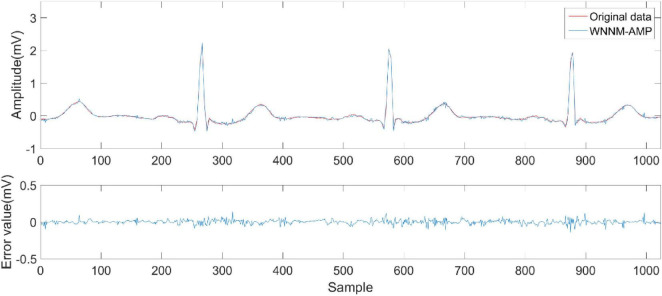
Reconstruction results of WNNM-AMP algorithm.

**FIGURE 9:**
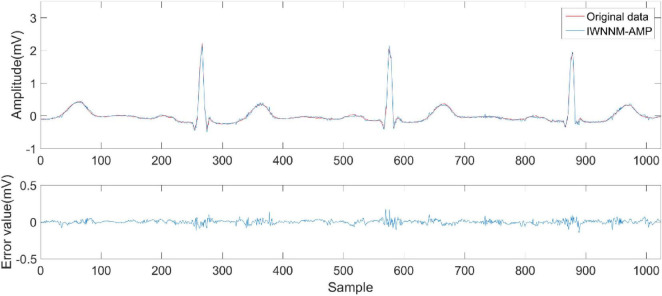
Reconstruction results of IWNNM-AMP algorithm.

Figures 6–9 compare the reconstruction results of the IWNNM-AMP algorithm with three other algorithms from the subjective visual perspective under the condition of an additional measurement noise standard deviation of 20 and compression ratio CR = 5.12. Through the comparison of reconstruction results, we found that the NLM-AMP and LRA-SVD-AMP algorithms have poor reconstruction quality, indicating that these algorithms perform poorly in reconstructing measured values with noise. Compared to the WNNM-AMP algorithm, the IWNNM-AMP algorithm has better visual reconstruction effects and superior detail reconstruction capabilities.

#### 4.3.4 Complexity analysis

Assuming the noisy signal *x* ∈ *R^N^* and the signal block *q^i^* ∈ *R^d^*, the time complexity of calculating all similar blocks is *o*((*N* − *d* + 1)^2^). Considering that the time complexity is too large, the algorithm defines a window range *wSize* for signal block *q^i^* and calculates the similarity between signal block *q^i^* and the signal blocks within window range *wSize*. This not only reduces the time complexity, but also because similar blocks to a signal block are usually located around this signal block. Performing low-rank approximation on groups of similar signal blocks, the algorithm complexity is *o*(*d*^3^).

Table 3 compares the running time of six reconstruction algorithms at different sampling rates. In the case of no measurement noise, with a signal length of 1,024 and 30 iterations, the running time of six algorithms at different sampling rates is compared, as shown in Table 3. The computation time of WNNM-AMP and IWNNM-AMP algorithms is longer than that of NLM-AMP algorithm. This is because it takes a certain amount of time to search for similar signal blocks. The running time can be reduced by limiting the search range. The running time of the IWNNM-AMP algorithm is basically the same as that of the WNNM-AMP algorithm. This is because the weight value of the signal block calculated by the IWNNM-AMP algorithm is obtained in the process of low-rank matrix approximation, and the computational complexity is not increased compared with the WNNM-AMP algorithm.

**TABLE 3:** Relative values of running time of six algorithms.

重构算法	压缩率								
	1.03	1.30	1.28	1.46	1.70	2.04	2.56	3.41	5.12
EMDD-AMP	3.01	2.95	2.81	2.75	2.51	2.17	1.80	1.55	1.46
WAVE-AMP	0.49	0.46	0.44	0.40	0.37	0.34	0.32	0.29	0.27
NLM-AMP	0.52	0.49	0.47	0.46	0.44	0.40	0.36	0.33	0.31
LRA-SVD-AMP	0.58	0.55	0.54	0.52	0.49	0.47	0.43	0.40	0.38
WNNM-AMP	0.73	0.70	0.67	0.65	0.62	0.59	0.57	0.54	0.52
IWNNM-AMP	0.72	0.70	0.67	0.65	0.63	0.59	0.58	0.54	0.51

#### 4.3.5 EEG signal reconstruction performance

**FIGURE 10:**
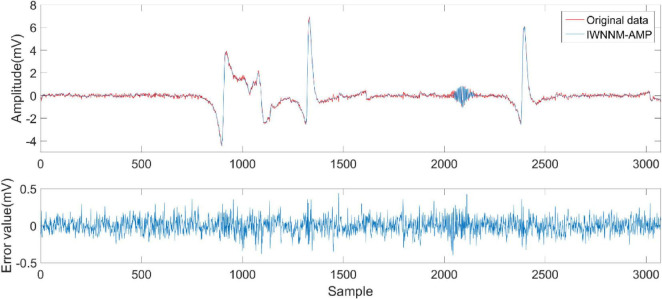
Reconstruction results of EEG signal.

This experiment used the NT9200 digital electroencephalogram (EEG) recorder to record EEG signals, with 32-channel recording electrodes placed according to the international standard 10–20 system. The sampling rate was 256 Hz, the recording duration was approximately half a minute, and further filtering was applied (notch filter at 50 Hz and bandpass filter 4–45 Hz). The collected data is compressed to verify the reconstruction performance of the IWNNM-AMP algorithm. Figure 10 shows the reconstruction effect of the EEG signal when the compression rate CR = 5.12 and the number of iterations is 30. The experimental results show that under high compression rate, the reconstruction error of the EEG signal of the IWNNM-AMP algorithm is between ± 0.5, and it has good reconstruction performance.

### 4.4 Results and discussion

Based on the above experimental results, the IWNNM-AMP algorithm proposed in this paper shows the best reconstruction performance under different compression ratios and noise conditions, achieving the lowest PRD and RMSE values. This indicates that the IWNNM-AMP algorithm can achieve higher accuracy in reconstructing electrocardiogram signals compared to other reconstruction algorithms. At the same time, in high compression ratio reconstructions, the PRD and RMSE values of the IWNNM-AMP algorithm are lower than those of the comparison algorithms, indicating that the IWNNM-AMP algorithm can reconstruct the electrocardiogram signals using less effective information, reducing the data volume for signal sampling and transmission. From a subjective visual perspective, the IWNNM-AMP algorithm has smaller reconstruction errors and better visual effects, with better ability to reconstruct details.

## 5 Conclusion

Non-local self-similarity is an important prior in signal denoising algorithms. In the presence of noise, dissimilar signal blocks may be grouped together, and dissimilar block groups do not have low rank properties. Therefore, it is inappropriate for the WNNM algorithm to average the signal blocks to obtain the denoised signal. Theoretically, the greater the similarity between similar blocks, the lower the rank of the matrix and the better the denoising effect. Therefore, this paper proposes a denoising approximate message passing reconstruction algorithm based on an IWNNM algorithm. In the denoising process, weighted averaging is used instead of direct averaging. Different weight values are assigned to the signal blocks according to the size of the rank of the similar block group. The smaller the rank of similar block groups, the more similar the signal blocks, and the larger the weight coefficient; the larger the rank of similar block groups, the smaller the weight coefficient.

The experimental verification selected the data set of MIT-BIH arrhythmia database. The results show that the proposed IWNNM-AMP algorithm can achieve better reconstruction performance than the WNNM-AMP algorithm, and in the reconstruction with high compression ratio, the PRD value is reduced by 0.17∼4.56, the RMSE value is reduced by 0∼0.013, and the P-SNR value is increased by 0.12∼2.70.

One drawback is that wearable devices may introduce noise during the actual collection of physiological signals, including baseline drift noise, power frequency interference, high-frequency electromyographic interference, and motion artifacts. The frequency range of motion artifacts overlaps with the frequency range of physiological signals, making it difficult to distinguish which frequency components belong to the heart rate and which belong to motion artifacts interference. Therefore, further research is needed on how to remove motion artifacts noise on the basis of blind denoising.

## Data Availability

The original contributions presented in this study are included in this article/supplementary material, further inquiries can be directed to the corresponding author.

## References

[B1] Aharon M. Elad M. Bruckstein A. (2006). K-SVD: an algorithm for designing overcomplete dictionaries for sparse representation. *IEEE Trans. Signal Process.* 54 4311–4322. 10.1371/journal.pone.0169663 28103283 PMC5245881

[B2] Balouehestani M. Raahemifar K. Krishnan S. (2013). “New sampling approach for wireless ECG systems with compressed sensing theory,” in *Proceeings of the Medical Measurements and Applications (MeMeA)*, (Piscataway, NJ: IEEE Press), 213–218. 10.1109/EMBC.2013.6610602

[B3] Dabov K. Foi A. Katkovnik V. Egiazarian K. (2007). Image denoising by Sparse 3-D transform-domain collaborative filtering. *IEEE Trans. Image Process.* 16 2080–2095. 17688213 10.1109/tip.2007.901238

[B4] Donoho D. Maleki A. Montanari A. (2009). Message-passing algorithms for compressed sensing. *Proc. Natl. Acad. Sci. U S A.* 106 18914–18919. 19858495 10.1073/pnas.0909892106 PMC2767368

[B5] Guo Q. Zhang C. Zhang Y. Liu H. (2016). An efficient SVD-based method for image denoising. *IEEE Trans. Circuits Syst. Video Technol.* 26 868–880.

[B6] He L. Chen H. Carin L. (2010). Tree structured compressive sensing with variational bayesian analysis. *IEEE Signal Processing Lett.* 17 233–236.

[B7] Hill P. Kim J. Basarab A. Kouame D. Bull D. Achim A. (2016). “Compressive imaging using approximate message passing and a cauchy prior in the wavelet domain,” in *Proceeings of the IEEE International Conference on Image Processing*, (Piscataway, NJ: IEEE Press).

[B8] Li Z. Huang H. Misra S. (2017). Compressed sensing via dictionary learning and approximate message passing for multimedia internet of things. *IEEE Internet Things J.* 4 505–512.

[B9] Liu X. Xia S. Fu F. (2017). “Reconstruction guarantee analysis of basis pursuit for binary measurement matrices in compressed sensing,” in *Proceeings of the IEEE International Symposium on Information Theory Proceedings*, (Piscataway, NJ: IEEE Press), 474–478.

[B10] Liu Z. Wen J. He T. (2010). Application of wavelet threshold de-noising technique in the gearbox vibration signal processing. *Modern Manuf. Eng.* 4 75–78.

[B11] Metzler C. Maleki A. Baraniuk R. (2016). From denoising to compressed sensing. *IEEE Trans. Inf. Theory* 62 5117–5144.

[B12] Nguyen N. Needell D. Woolf T. (2017). Linear convergence of stochastic Iterative greedy algorithms with sparse constraints. *IEEE Trans. Inf. Theory* 63 6869–6895.

[B13] Ramani S. Blu T. Unser M. (2008). Monte-carlo sure: a black-box optimization of regularization parameters for general denoising algorithms. *IEEE Trans. Image Process.* 17 1540–1554. 10.1109/TIP.2008.2001404 18701393

[B14] Scetbon M. Elad M. Milanfar P. (2021). Deep K-SVD denoising. *IEEE Trans. Image Process.* 30 5944–5955. 34166193 10.1109/TIP.2021.3090531

[B15] Shoaran M. Afshari H. Schmid A. (2014). “A novel compressive sensing architecture for high-density biological signal recording,” in *Proceeings of the 2014 IEEE Biomedical Circuits and Systems Conference (BloCAS),.13-16*, (Piscataway, NJ: IEEE).

[B16] Som S. (2012). Compressive imaging using approximate message passing and a markov-tree prior. *IEEE Trans. Signal Processing* 60 3439–3448. 10.3390/s20164609 32824410 PMC7472005

[B17] Tan J. Ma Y. Baron D. (2015). Compressive imaging via approximate message passing with image denoising. *IEEE Trans. Signal Processing* 63 2085–2092.

[B18] Wang X. Liang J. (2015). “Multi-resolution compressed sensing reconstruction via approximate message passing,” in *Proceeings of the 2015 IEEE International Conference on Image Processing, Quebec Canada*, (Piscataway, NJ: IEEE Press).

[B19] Xi X. Wu H. Luo Z. (2014). Denoising method of the sEMG based on EMD autocorrelation. *Chin. J. Sci. Instrument* 35 2494–2500.

[B20] Xu M. Hu D. Luo F. Liu F. Wang S. Wu W. (2021). Limited angle X ray CT reconstruction using image gradient L0 norm with dictionary learning. *Proc. IEEE Trans. Radiation Plasma Med. Sci.* 5 78–87.

[B21] Xue S. Yin C. Su Y. Liu Y. Wang Y. Liu C. (2020). Airborne electromagnetic data denoising based on dictionary learning. *Appl. Geophys.* 17 306–313.

[B22] Zhang Z. Jung P. Makeig S. (2013). Compressed sensing for energy-efficient wireless telemonitoring of noninvasive fetal ECG via block sparse Bayesian learning. *Biomed. Eng.* 60 300–309. 10.1109/TBME.2012.2226175 23144028

